# Cefdinir-Induced Liver Injury: A Case Report

**DOI:** 10.7759/cureus.81523

**Published:** 2025-03-31

**Authors:** Muhammad Areeb Ashfaq, Hajira Z Malik, Leena Patel, Cesar Moreno

**Affiliations:** 1 Internal Medicine, University of South Alabama, Mobile, USA; 2 Cardiology, University of South Alabama, Mobile, USA; 3 Gastroenterology, University of South Alabama, Mobile, USA

**Keywords:** acute hepatotoxicity, cefdinir, drug-induced liver injury (dili), elevated liver-associated enzymes, hepatocellular adaptive changes

## Abstract

Cephalosporins are very rarely known to cause drug-induced liver injury (DILI). We present the case of a healthy 40-year-old female who developed DILI after completing a course of cefdinir, a third-generation cephalosporin, to treat a urinary tract infection. A 40-year-old female with no past medical history presented to the emergency department with a chief complaint of epigastric pain and jaundice for the last four days. A workup at an urgent care revealed hyperbilirubinemia and elevated liver enzymes, prompting this visit. She had completed a five-day course of cefdinir for a urinary tract infection three weeks prior to her visit. Physical examination revealed a vitally stable patient with scleral icterus and no other positive findings. Laboratory workup was significant for total bilirubin 4.3 mg/dL, direct bilirubin 0.7 mg/dL, ALP 130 unit/L, ALT 546 unit/L, and AST 213 unit/L. Serum hepatitis panel, ferritin, ceruloplasmin, alpha-1 antitrypsin, and autoimmune workup were within normal limits. Ultrasound and CT of the abdomen and magnetic resonance cholangiopancreatography did not reveal any pathology. Liver biopsy demonstrated hepatocellular adaptive changes and mild bile duct epithelial damage, suggestive of DILI. This case highlights the importance of obtaining a thorough medical history, including recent medication use, drug dose, and time of symptom onset, for a patient presenting with elevated liver enzymes. This case also emphasizes keeping a broad list of differential diagnoses when managing patients with elevated liver enzymes.

## Introduction

Drug-induced liver injury (DILI) is an uncommon hepatic condition occurring in response to the use of medications, illegal drugs, herbal products, or dietary supplements [1]. DILI is estimated to be between one and 20 cases per 100,000 persons exposed in a year, and it carries a mortality rate of up to 10% [1,2]. Presentation of DILI may vary from asymptomatic increases in liver-associated enzymes to cholestatic and/or hepatocellular jaundice, acute liver failure, or chronic hepatitis [1]. In the United States, DILI accounts for nearly 10% of the total cases of acute hepatitis and 50% of all cases of acute liver failure [2,3].

The mechanism of induction of liver injury is a complex interplay between several host, drug, and environmental factors and is multifactorial. Acetaminophen is the most common drug implicated in DILI [4]. While several classes of antibiotics are commonly known to cause DILI, cephalosporins are rarely known to cause hepatotoxicity. To the best of our knowledge, there is only one reported case of cefdinir-induced hepatotoxicity previously reported in PubMed in the last 20 years [5].

We present the case of a healthy 40-year-old female who developed DILI after completing a course of cefdinir, a third-generation cephalosporin, to treat a urinary tract infection.

## Case presentation

Our patient is a 40-year-old Caucasian female with no past medical history who presented to the emergency department with the chief complaint of epigastric pain and jaundice for four days. She had blood work done at an urgent care and was found to have hyperbilirubinemia and elevated liver enzymes, prompting this visit. She denied nausea, loss of appetite, fever, or other gastrointestinal symptoms. The patient did not smoke, drink alcohol, or use illicit drugs. She had not been on any herbal or over-the-counter medications. The patient further stated that she had completed a five-day course of cefdinir 300 mg twice a day for a urinary tract infection three weeks ago with resolution of symptoms. At the time of presentation, she was vitally stable with a temperature measuring 36.8°C, heart rate 88 bpm, blood pressure 123/82 mmHg, and respiratory rate 17. On physical examination, the only notable finding was scleral icterus. Laboratory workup on presentation was significant for raised total bilirubin 4.3, direct bilirubin 0.7, ALP 130, ALT 546, and AST 213. CBC was unremarkable. Hepatitis panel, ferritin, ceruloplasmin, alpha-1 antitrypsin, acetaminophen level, coagulation profile, lipid panel, glucose-6-phosphate dehydrogenase level, and autoimmune workup were within normal limits. Ultrasound and CT of the abdomen did not reveal any pathology. The patient was conservatively managed during the hospital stay with adequate pain control. Gastroenterology was consulted, and a percutaneous liver biopsy was performed, which demonstrated hepatocellular adaptive changes and mild bile duct epithelial damage, suggestive of DILI. There was no necrosis, and iron and periodic acid-Schiff-diastase stains were negative. Magnetic resonance cholangiopancreatography was also performed soon after, which was unremarkable, further completing the workup and excluding other causes of liver injury and liver disease. The patient remained stable throughout the hospital stay and was counseled to avoid all hepatotoxic medications. She was discharged in stable condition. She followed up in an outpatient setting with gastroenterology, and her repeat laboratory workup in 10 days showed improvement (Table 1).

**Table 1:** Pertinent laboratory investigations

	Reference ranges	On arrival	Two days later	10 days later
Alanine transaminases unit/L	16-63	546	352	68
Aspartate aminotransferase unit/L	15-37	213	87	19
Total bilirubin mg/dL	0.0-1.0	4.3	3.0	1.7
Alkaline phosphatase unit/L	45-117	130	112	82

## Discussion

DILI accounts for over half of the cases of acute liver injury across most Western countries. If hepatocellular jaundice is present, mortality may reach up to 10% [6]. It is largely diagnosed based on exclusion, with other prominent causes of acute liver injury ruled out, as was done with our patient, including viral hepatitis, autoimmune conditions, and DILI secondary to well-known medications, including amoxicillin-clavulanic acid. Clinical classification of DILI includes hepatocellular, cholestatic, and mixed injury based on the pattern of liver injury and enzyme elevation [7]. Our patient had hepatocellular injury with jaundice as evidenced by the R-factor calculated from her labs on arrival. R-factor is a calculator that assists physicians in differentiating cholestatic from hepatocellular injury. Based on histologic findings, the classifications are hepatitis, cholestasis, and steatosis [6,8]. Our patient’s liver biopsy was evidence of hepatocellular damage.

LiverTox, an NIH-sponsored website, keeps track of all drugs with at least one case report about causing DILI [9]. Acetaminophen remains the most common drug to cause liver injury [4]. Antibiotics are another common source of DILI. A recent single US center experience reported antibiotics as the class of drugs most frequently implicated in non-fulminant drug-induced hepatitis [10]. Amoxicillin/clavulanic acid, minocycline, nitrofurantoin, trimethoprim-sulfamethoxazole, and trovafloxacin were the most frequently implicated antibiotics. Across some other countries, antibiotics are recognized as the most frequent cause of DILI [11].

Cefdinir does not routinely require dose adjustment for any level of liver impairment (Child-Pugh score). It binds to penicillin-binding proteins, leading to inhibition of bacterial cell wall synthesis. It has minimal hepatic metabolism and is primarily excreted in the urine. Common side effects include diarrhea, nausea, and skin rashes [12].

However, DILI caused by cephalosporins is very rarely reported. Cefdinir is an oral third-generation cephalosporin, effective against numerous Gram-positive and Gram-negative bacteria. Most common side effects include gastrointestinal distress, nausea, or vomiting. There is only one recorded case of cefdinir-induced hepatotoxicity in 2008 [5]. In that case, the presentation was similar to our patient with jaundice and elevation of liver enzymes. However, the patient required treatment with steroids due to progressively worsening jaundice, whereas our patient gradually recovered with symptomatic management.

## Conclusions

This case suggests the addition of cefdinir to the ever-growing list of medications causing DILI. It also highlights the importance of obtaining a thorough medical history, including recent medication use, drug dose, and time of symptom onset, for a patient presenting with elevated liver enzymes. It is important to exclude other more common causes of liver injury. Liver biopsy is not typically obtained if there has been exposure to a drug widely known to cause DILI. However, as in our case, when the diagnosis is uncertain or the drug in question is not commonly known to cause liver injury, a liver biopsy needs to be performed for a definitive diagnosis.
